# Long non-coding RNAs as novel players in β cell function and type 1 diabetes

**DOI:** 10.1186/s40246-017-0113-7

**Published:** 2017-07-24

**Authors:** Aashiq H. Mirza, Simranjeet Kaur, Flemming Pociot

**Affiliations:** 1 CPH-DIRECT, Department of Pediatrics, Herlev University Hospital, Herlev Ringvej 75, DK-2730 Herlev, Denmark; 2 Faculty of Health and Medical Sciences, University of Copenhagen, Copenhagen, Denmark; 3 Center for non-coding RNA in Technology and Health, University of Copenhagen, Copenhagen, Denmark

**Keywords:** Long non-coding RNAs, Type 1 diabetes, Enhancers, Regulatory elements, 3D genome architecture

## Abstract

**Background:**

Long non-coding RNAs (lncRNAs) are a sub-class within non-coding RNA repertoire that have emerged as crucial regulators of the gene expression in various pathophysiological conditions. lncRNAs display remarkable versatility and wield their functions through interactions with RNA, DNA, or proteins. Accumulating body of evidence based on multitude studies has highlighted the role of lncRNAs in many autoimmune and inflammatory diseases, including type 1 diabetes (T1D).

**Main body of abstract:**

This review highlights emerging roles of lncRNAs in immune and islet β cell function as well as some of the challenges and opportunities in understanding the pathogenesis of T1D and its complications.

**Conclusion:**

We accentuate that the lncRNAs within T1D-loci regions in consort with regulatory variants and enhancer clusters orchestrate the chromatin remodeling in β cells and thereby act as cis/trans-regulatory determinants of islet cell transcriptional programs.

## Background

Type 1 diabetes (T1D) is a chronic immune-mediated disease resulting from selective destruction of insulin-producing pancreatic islet β cells. A complex interplay between several environmental and genetic risk factors contribute to the onset of T1D [1, 2]. Defects in both immune system and β cells play an active role in T1D pathogenesis [3]. In recent years, efforts have been accelerated to gain insights into the molecular mechanisms of pathogenesis of T1D and to determine how genetic loci contribute to the T1D risk [1, 4]. Based on genome-wide association studies (GWAS), currently more than 50 genomic risk loci have been identified for T1D [2, 5–7]. Approximately, 50% of the genetic risk for T1D is known to reside within the human leukocyte antigen (HLA) region; however, other non-HLA disease susceptibility loci have been identified based on their direct influence on the risk. Some of the well-established candidate genes in the non-HLA risk loci including *INS* (11p15), *CTLA4* (2q33), *PTPN22* (1p13), *PTPN2* (18p11), *ERBB3* (12q13), *IL2RA* (10p15), and *IFIH1* (2q24) have been associated with immune response, insulin expression, and β cell function [1, 4, 8]. The risk alleles for several T1D susceptibility genes are not exclusively confined to T1D but have been shown to confer risk in other prevalent autoimmune disorders, including multiple sclerosis (MS), systemic lupus erythematosus (SLE), and rheumatoid arthritis (RA) [7]. Furthermore, most of these risk variants are located in non-coding genomic regions including long non-coding RNAs (lncRNAs) and are enriched in distal regulatory elements such as enhancers and promoters. Non-coding variants affecting regulatory elements have the potential to perturb chromatin folding leading to mis-expression of the target gene. These facts suggest that the regulatory landscape of human genome plays an important role in pathology of a disease and newer approaches are needed to identify putative regulatory risk variants affecting gene regulation and immune function.

Recent advances in our understanding of lncRNA biology has offered new perspectives on gene regulation and has allowed us to unveil the regulatory potential of these versatile molecules in a spectrum of biological processes and pathologies, including autoimmune and inflammatory disorders. High-throughput technologies such as ChIP-seq and chromosome conformation capture techniques have also opened new possibilities to investigate in detail potential regulatory roles of non-coding genome in gene regulation and 3D chromatin folding. In this review, we discuss the recent discoveries in the field of lncRNAs, regulatory elements, 3D genome architecture, and their implications for T1D and β cell function. We further highlight the role of active enhancers associated with T1D–loci lncRNAs and protein-coding genes in regulating β cell gene expression programs through both *cis*- and *trans*-regulatory mechanisms involving structural remodeling of chromatin in human islets.

## Main text

### lncRNAs in T1D and other immune-mediated diseases

**Fig. 1:**
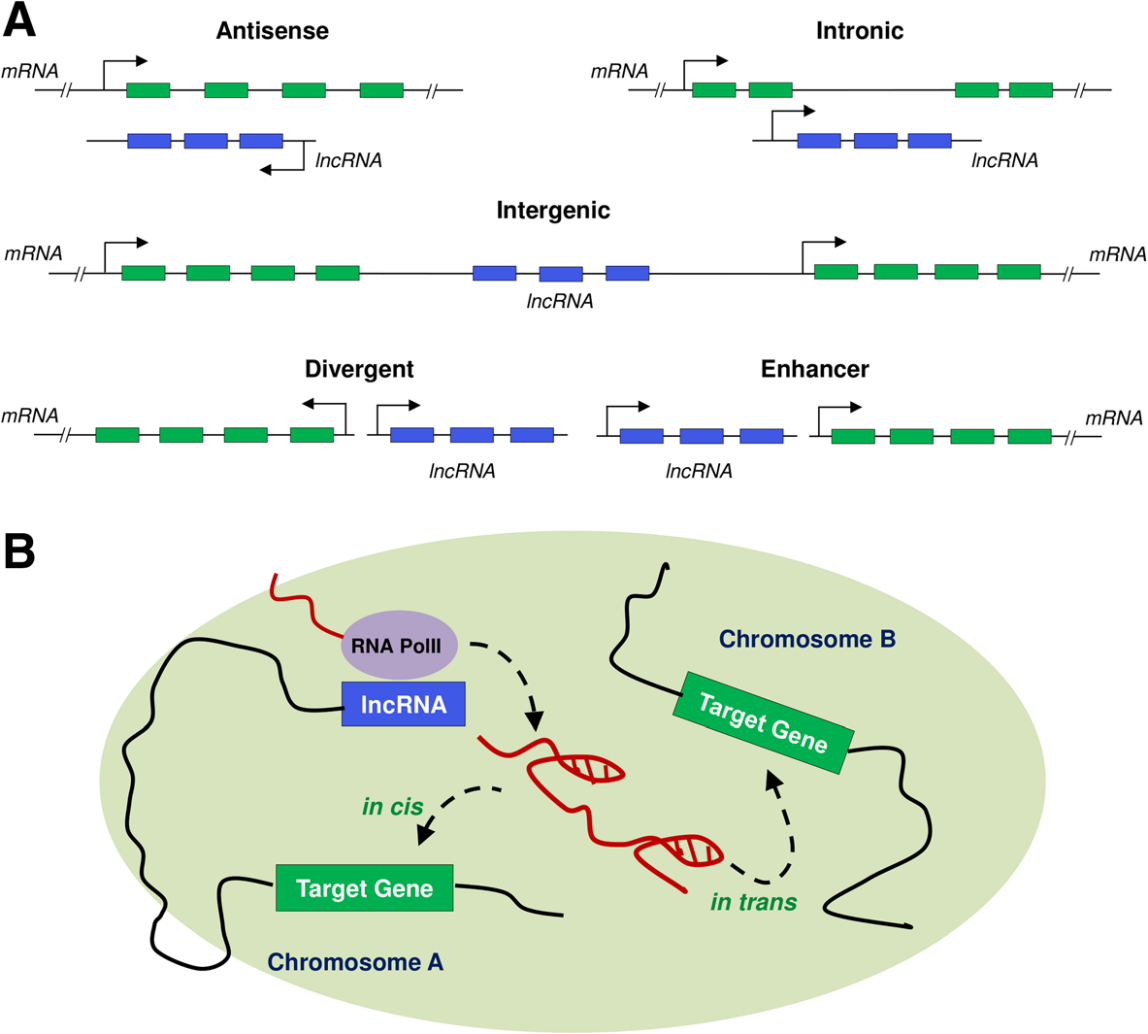
Bio-types of lncRNAs and enhancer-derived lncRNA function. **a** Different bio-types of lncRNAs based on their genomic location include antisense, intergenic, intronic, divergent and enhancer-derived lncRNAs. lncRNAs are depicted in *blue*, while protein-coding genes are shown in *green*. **b** The postulated role of enhancer-derived lncRNAs for both *cis-* and *trans*-mediated regulation of target genes is shown via chromatin loop formation. Figure modified from Ref. [10, 11]

lncRNAs are non-coding RNAs that are more than 200 nucleotides in length, and are capped, polyadenylated, and spliced like their well-characterized “cousins,” protein-coding transcripts, with one exception; lncRNAs do not code for proteins [9]. Most of the lncRNAs are expressed in a cell-specific manner and are usually expressed in lower abundance than the protein-coding transcripts. In terms of genomic location, lncRNAs have been often categorized as long intergenic non-coding RNAs (lincRNAs), intronic lncRNAs, antisense lncRNAs, divergent lncRNAs and enhancer-derived lncRNAs (lncRNAs arising from enhancer-like regions) [10–12] (Fig. 1). lncRNAs have emerged as important players of gene regulation and have been implicated in various human pathologies [13]. lncRNAs regulate various cellular and biological processes including heterochromatin formation, histone modifications, DNA methylation targeting, and gene silencing [14, 15]. The lncRNA-recruited regulatory complexes orchestrate development and differentiation of various immune cell lineages and actively regulate expression programs within these cells.

Multiple studies have highlighted the potential roles of lncRNAs in pancreatic islets and T1D pathogenesis [10, 16, 17]. Based on transcriptome profiling studies of islets and β cells, more than 1000 islet-specific lncRNAs have been identified in both human and mouse islets [18, 19]. The ability of lncRNAs to regulate gene expression and cell-specific identity provides an exciting opportunity to advance our understanding of T1D pathogenesis. Table 1 lists examples of lncRNAs that have been implicated in β cell function and T1D. lncRNA *MALAT1* has been associated with diabetes-induced microvascular dysfunction in STZ-induced diabetic rats and db/db mice [20]. Knockdown of *MALAT1* prevents the hyper-proliferation of retinal endothelial cells through p38 MAPK signaling and might serve as a potential target for anti-angiogenic therapy for diabetic retinopathy. lncRNA *MEG3* has been associated with paternally inherited risk of T1D [21] and its downregulation affects insulin synthesis and secretion in mouse β cells [22]. The knockdown of *trans*-acting islet-specific lncRNA *HI-LNC25* (*LINC01370*) in mature β cells resulted in downregulation of *GLIS3* gene [18]. *GLIS3* encodes an islet transcription factor (TF) and is a candidate gene for both type 1 and type 2 diabetes. Another islet-specific lncRNA *βlinc1* (previously known as *HI-LNC15*) has been shown to be essential for proper specification and function of β cells [23]. Knockdown of *βlinc1* in mouse MIN6 cells and human insulin-producing EndoC-βH1 cells resulted in downregulation of several islet-specific TFs, including Nkx2.2, Pax6, and Mafb [23]. Also, deletion of *βlinc1* resulted in defective islet development and disrupted glucose homeostasis in the adult mice [23]. It is particularly intriguing that *βlinc1* specifically regulates three essential islet TFs (Nkx2.2, Pax6, and MafB) and additional β cell genes on chromosome 2, all of which are associated with endocrine development and maintaining islet morphology [23]. Nuclear-enriched β cell lncRNA *PLUTO (PLUT)* (previously known as *HI-LNC71*) regulates the transcription of *PDX1* gene which is a key pancreatic β cell transcriptional regulator [24]. *PLUT* encompasses a cluster of enhancers that make 3D contacts with the *PDX1* promoter in human islets and in human β cell line EndoC-βH1. Loss of *PLUT* was associated with downregulation of *PDX1* at both mRNA and protein levels in EndoC-βH1 cells, primary islet cells, and a similar effect was observed for the mouse lncRNA ortholog in mouse β cell line MIN6 [24]. Knockdown of lncRNA *TUNAR* (*HI-LNC78*) resulted in reduced insulin content and impaired glucose-stimulated insulin secretion in T antigen-excised EndoC-βH3 cells [24]. lncRNA *TUG1* is a highly conserved lncRNA in mammals and is highly expressed in mouse pancreatic tissues [25]. In mouse β cells, downregulation of *TUG1* as a consequence of hyperglycemia resulted in increased apoptosis and reduced insulin synthesis and secretion [25]. These findings suggest that *TUG1* may partially contribute to the impairment of β cell function and could therefore be implicated in diabetes pathogenesis.

**Table 1:** lncRNAs associated with β cell function and type 1 diabetes

lncRNAs	Function	Reference
*MEG3*	Regulates β cell identity and function via insulin production and apoptosis in mouse MIN6 cells and isolated mouse islets	You et al. 2016 [22]
*HI-LNC25*	Positively regulates *GLIS3* (which contains both T1D and T2D risk variants) in EndoC-βH1 human β cell line	Moran et al. 2012 [18]
*βlinc1 (HI-LNC15)*	Regulates β cell identity and function in mouse MIN6 cells and EndoC-βH1 human β cell line; also regulates its neighboring gene NKX2.2 (an islet transcription factor).	Arnes et al. 2016 [23]
*TUNAR (HI-LNC78)*	Knockdown of *TUNAR* leads to impaired glucose-stimulated insulin secretion in human islets	Akerman et al. 2017 [24]
*PLUT (HI-LNC71)*	Regulates transcription of *PDX1,* a key pancreatic β cell transcriptional regulator, in EndoC-βH1 cells, primary islet cells, mouse β cell line MIN6	Akerman et al. 2017 [24]
*MALAT1*	Upregulation of *MALAT1* is associated with microvascular dysfunction (diabetic retinopathy) in STZ-induced diabetic rats and db/db mice	Liu et al. 2014 [20]
*TUG1*	Downregulation of lncRNA TUG1 expression increased apoptosis and reduced insulin secretion in mouse β cells	Yin et al. 2015 [25]

In recent years, growing body of evidence has linked dysregulation of lncRNA expression to a spectrum of autoimmune disease [26, 27]. Hrdlickova and colleagues found enrichment of lincRNAs in autoimmune disease-associated loci in a subset of immune cells [28]. A number of studies describe the emerging role of lncRNAs in transcriptional regulation of inflammatory gene expression [29, 30]. For example, in human monocytes, lncRNA *THRIL* has been shown to interact with hnRNP-L and regulate the expression of *TNFα* [31]. Correspondingly, additional examples of lncRNAs involvement in inflammatory signaling cascades and regulation of innate immune responses includes (1) lncRNA *PACER* (*PACERR*) which has been shown to bind p50 subunit of *NFκB* and control the basal expression levels of *Cox2 (PTGS2)* [32]; (2) in primary human monocytes, knockdown of *NFκB* regulated, enhancer-RNA (eRNA) *IL1β-eRNA* and region of bidirectional transcription (RBT) IL1β-RBT46, mitigated bacterial lipopolysaccharide (LPS) pro-inflammatory cytokine IL1β induction and release [33]; (3) overexpression of natural antisense transcript anti-IL1β alters the chromatin structure around the IL1β promoter and consequently inhibits the IL1β expression [34, 35]. In a murine model, lncRNA *NeST* (Nettoie Salmonella pas Theiler’s; *cleanup Salmonella not Theiler’s*) was shown to epigenetically regulate the interferon-γ (IFN-γ) locus and control the susceptibility to Theiler’s virus and Salmonella infection [36, 37]. Together, these findings indicate that lncRNAs play etiological role in autoimmune diseases. lncRNAs have also been shown to play pivotal roles in the Toll-like receptor (TLR) signaling pathway. For example, when macrophages and dendritic cells (DCs) were stimulated with TLR ligands, the *lincRNA-Cox2A* was found to be highly inducible and also controlled the basal expression levels of interferon-stimulated genes (ISGs) and pro-inflammatory cytokines [29]. Intriguingly, pseudogenes lncRNAs have been identified to act as functional regulators of inflammatory signaling with their expression being actively regulated. Stimulation of mouse embryonic fibroblast cells by *TNFα* has been found to induce expression of lncRNAs. *Lethe*, a pseudogene lncRNA, has been shown to function as a novel negative regulator of *NF-κB*. *Lethe*, wields its regulatory function by binding directly RelA, a subunit of *NF-κB* heterodimeric complex, preclude *NF-κB* binding to the promoter regions of target genes [38]. Another pseudogene lncRNA Lnc-dendritic cell (*DC*) (*WFDC21P*) has been shown to be involved in monocyte to DC differentiation [39].

Systemic cell-mediated immunity is known to play a central role in the apoptotic β cell destruction that culminates in T1D. The T helper 17 (Th17) cells are known to protect mucosal barriers from opportunistic infections and are also associated with number of autoimmune inflammatory diseases. Like many other autoimmune diseases, T1D is also a T cell-mediated malady, and imbalance between the Th17 cells and T regulatory (Treg) has been implicated in development of the T1D [40, 41]. Recently, Huang et al. [42] demonstrated role of DEAD-box protein 5 (DDX5) as a binding partner of RORγt, a well-known ligand-regulated nuclear receptor that controls the differentiation of Th17 cells. Interestingly, DDX5 coordinates the transcription of selective Th17 genes through its interaction with RORγt, and it is also required for Th17 cell-mediated inflammatory diseases. The interaction between DDX5 and RORγt is dependent on the inherent RNA helicase activity of DDX5 and the binding of Rmrp, an evolutionarily conserved nuclear lncRNA. Furthermore, Rmrp was found mutated in patients with cartilage-hair hypoplasia, and corresponding mutation in Rmrp in mice resulted in altered chromatin interaction, and diminished interaction between the DDX5 and RORγt, and also downregulated expression of selective Th-17 genes [42].

These examples highlight the importance of lncRNAs in regulating gene expression in immune cells and underscore yet another layer of complexity in gene regulation. Future studies should be focused towards elucidating their molecular functions which in turn could provide crucial insights into novel mechanisms of gene regulation, autoimmune and inflammation-mediated disorders, including T1D.

### Genome-wide interactions between T1D SNPs, lncRNAs, enhancers, and other distal regulatory elements

More than 90% of disease-associated single-nucleotide polymorphisms (SNPs) are located within the non-coding regions of the genome such as promoters, enhancers, intergenic regions, and ncRNA genes [43]. The disease-associated SNPs have the potential to be regulatory in nature, particularly if they are significantly enriched in functional regulatory elements such as transcription factor binding sites (TFBSs), histone modification marks, DNase-I hypersensitive sites, and expression quantitative trait loci (eQTLs) [44, 45]. These disease-associated regulatory SNPs are also referred to as “functional SNPs” [44]. Approximately, 10% of the autoimmune disease-associated SNPs are present within lncRNAs and some of these SNPs are also known to act as *cis*-eQTLs [46]. It has been shown that 75% of the lincRNA *cis*-eQTLs specifically alter the expression of lincRNAs in a tissue-dependent fashion but does not affect the nearby protein-coding genes, and many of these *cis-*eQTLs SNPs are known to be associated with complex genetic diseases [47]. Since the expression of protein-coding genes can be regulated by lincRNAs either in *cis* [48] or *trans* [49] manner, this suggests a link between disease-associated SNPs within the non-coding regions with the regulation of protein-coding gene expression.

Distal regulatory elements such as enhancers, locus control regions (LCRs), and insulators are highly abundant in the human genome and play an important role in transcriptional control. These elements represent the primary mechanism by which cell and developmental specific gene expression is accomplished. Enhancers are regulatory sequences that can activate gene expression independent of their proximity to their target genes in a tissue-specific manner [50]. Multiple enhancer elements arrayed over large regions can synergistically regulate the expression of individual genes or gene clusters by altering the TF binding and chromatin states [51, 52]. Additionally, multiple polymorphisms in linkage disequilibrium (LD) impact clusters of enhancer elements active in the same cell type and cooperatively contribute to altered expression of their gene targets [53]. The multiple enhancer variants within a given locus typically target the same gene which results in either gain- or loss-of-function [53]. Additionally, the genes associated with multiple enhancer variants encode proteins that are often functionally related and enriched in common pathways. Recently, several methods have been developed for genome-wide prediction of enhancers primarily based on chromatin marks such as H3K4 monomethyl (H3K4me1) and H3K27 acetyl (H3K27ac) modifications, bi-directional transcription, and binding of p300 [54–57]. The underlying mechanism for enhancer function has been suggested to involve formation of long-range chromatin loops, bringing enhancers and promoters into proximity and allowing interaction of the necessary co-transcriptional factors [58, 59]. Formation of chromatin loops occurs between two distant genomic sequences that are brought in close vicinity by protein complexes and are assumed to be chemically cross-linked. Various chromosomal conformation capture techniques such as 3C (chromosome conformation capture) [60], 4C (circular chromosome conformation capture) [61], 5C (chromosome conformation capture carbon copy) [62], ChIA-PET (Chromatin Interaction Analysis with Paired-End-Tag sequencing) [63], and Hi-C [64] have been used to detect genome-wide chromosome interactions. Examples of long-range interactions within mammalian gene loci include the locus control region (LCR) and β-globin promoter [65, 66]; the α-globin gene cluster in erythroid cells [67]; the TH2 and MHC loci in T cells [68, 69]; and the imprinted gene clusters Dlx5, Dlx6 [70], and H19-Igf2 [71–73]. Additional example of long-range chromatin loop mediated interaction among regulatory elements on different chromosomes has been observed at the IFNγ and TH2 cytokine loci [74]. The transcriptional regulation of IL-21 gene at the chromatin level was recently uncovered to be mediated through long-range chromatin interaction in CD4+ T cells. IL-21, a pro-inflammatory cytokine with pleiotropic effects, is strongly associated with autoimmunity and inflammation and regulates various immune responses**.** A study by Park et al. showed that a distal enhancer element within an evolutionary conserved non-coding sequence 49 kb upstream of the IL-21 can upregulate IL-21 gene expression in a STAT3- and NFAT-dependent manner [75]. Stimulation of CD4+ T cells with IL-6 leads to the recruitment of STAT3 to the IL-21 promoter and the distal enhancer region, bringing them in close spatial proximity. As a consequence, this long-range interaction between the promoter and distal enhancer region dependent on IL-6/STAT3 signaling pathway alters the chromatin configuration dynamically, and controls the expression of IL-21 in CD4+ T cells [75].

**Fig. 2:**
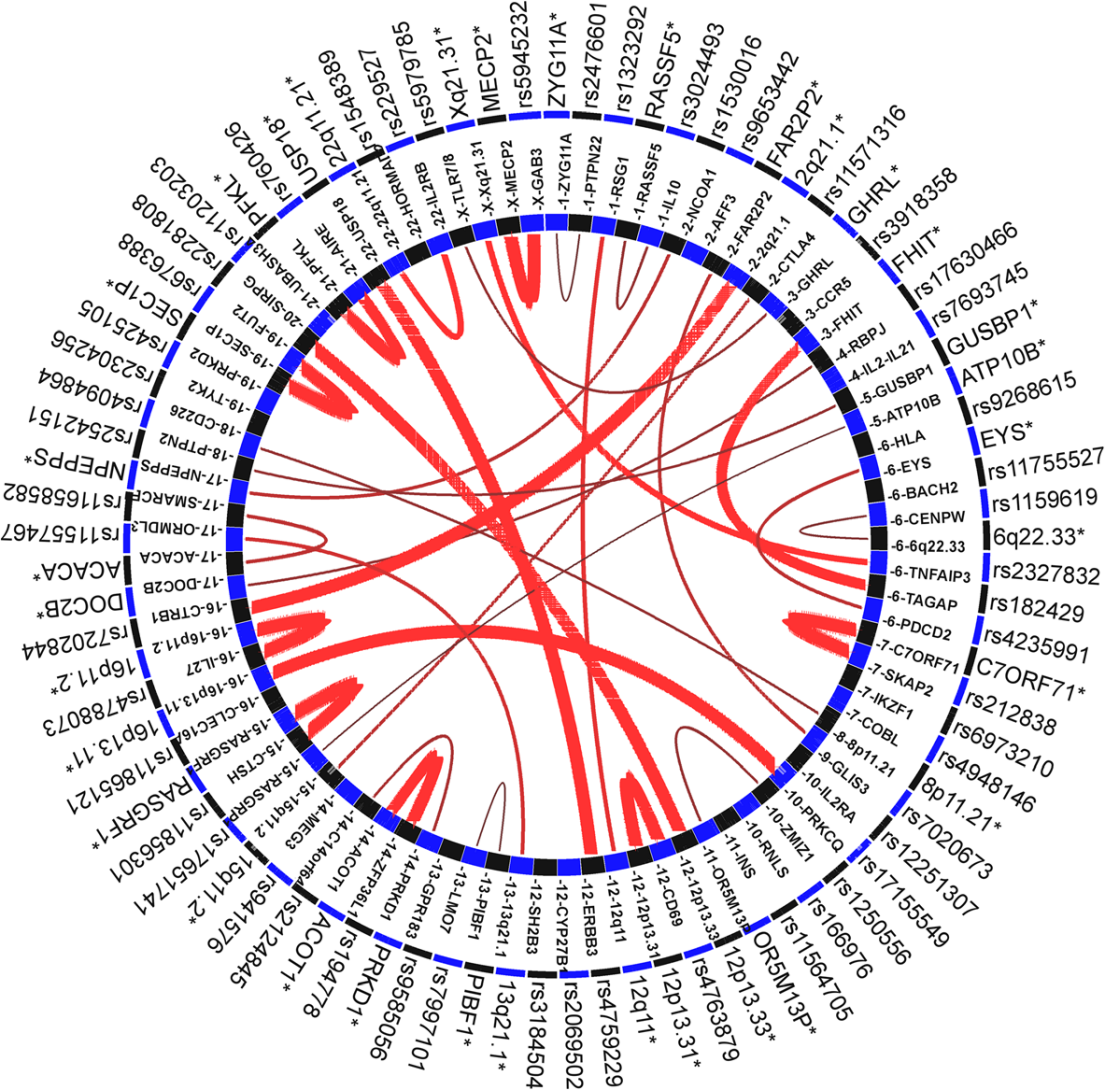
Distal regulatory elements and potential functional T1D SNPs in T1D susceptibility loci. The *inner circle* represents T1D susceptibility genes and genomic loci and the *outer circle* lists the regulatory variants and distal interaction regions. The distal interactive elements are denoted by an *asterisk* which includes enhancer elements and lncRNAs. The *red lines* indicate long-range chromosome interaction signals to another locus and the intensity of interaction is represented by the width of the *red lines*

Based on published genome-wide chromosome conformational capture datasets from various cell-lines and T1D associated SNPs we identified physical interactions between distant regulatory regions in T1D loci. Figure 2 shows an interactive map of T1D loci highlighting the physical interactions between distal regulatory elements and potential functional T1D SNP at each locus**.** T1D risk SNPs and SNPs in LD (r2 > 0.8, CEU HapMap3 population) were selected and scored based on the original GWAS signal, long range chromosome interactions, overlap with chromatin marks, epigenetic modifications and sequence motifs from various ENCODE cell lines [2, 76–78]. The top most scoring SNP for each region was inferred as the most significant functional SNP and selected for plotting along with the most significant distal chromosomal interaction signal. As an example, in *ERBB3* locus, SNP rs4759229 qualified as the most significant variant. rs4759229 is in perfect LD with T1D risk SNP rs2292239, overlaps a known enhancer region and has a long range interaction signal with an antisense lncRNA *AC008079.1* (Ensembl ID: ENSG00000187979) located at USP18 locus on chromosome 22. In a recent study, we proposed that the *ERBB3* SNP rs2292239 and its proxy SNPs in perfect LD rs3741499 and rs4759229 are putatively functional based on the overlapping open chromatin marks, TFBs and DNase I hypersensitivity peaks [79]. We further showed that SNP rs4759229 overlaps a known enhancer element and is in the vicinity of several lncRNA transcripts overlapping *ERBB3* locus. The potential functional T1D SNPs within known T1D candidate genes such as *GLIS3*, *ERBB3*, *CTRB1*, *CTSH*, *FUT2*, *IL27*, *SKAP2*, *TNFAIP3* and *PTPN2* had highly significant distal chromosomal interactions including enhancers and lncRNAs that are worthy of specific laboratory investigations (Fig. 2). Intriguingly, the impact of interactions between T1D SNPs and enhancer associated lncRNAs on transcription and ultimately on T1D risk remains to be seen. Based on the above evidence we postulate that the T1D SNPs mapping to enhancer associated lncRNAs could potentially alter the expression of their gene targets through enhancer mediated interactions and thereby significantly impact β cell gene expression.

Many lncRNAs have been reported to be expressed from the enhancer regions that are produced by activity-dependent RNA polymerase II binding of specific enhancers [80]. The expression levels of these enhancer associated lncRNAs positively correlate with the expression of neighboring protein-coding genes, i.e., depletion of enhancer associated lncRNAs led to decreased expression of their neighboring protein-coding genes [48, 80]. In addition, enhancers overlapping lncRNAs have higher H3K4me3/H3K4me1 ratios as compared to enhancers that do not overlap lncRNAs [81]. The enrichment of H3K4me3 marks (which are also associated with active promoters) points towards strong transcriptional capabilities of overlapping lncRNAs. lncRNAs transcribed from active enhancers have been identified as important players in mediating enhancer function [82]. Enhancers associated lncRNA play roles in important physiological processes and influence the activation of protein coding and non-coding genes in both *cis* and *trans* mediated mechanism. For example, lncRNA *NEST* is involved in *cis*-activation of the neighboring interferon γ locus, whereas lncRNA *Jpx* regulates *trans*-activation of another lncRNA, *XIST*, (which is critical for X inactivation) [36, 83]. While acting in *trans*, enhancer associated lncRNAs act over long distances by long range chromatin loop mediated interactions and activate transcription at distal promotors. It has also been suggested that bridging factors such as Mediator/Cohesin complex and enhancer associated lncRNAs are involved in establishment of chromatin looping between the lncRNAs and their regulated distal promotors [84]. Knockdown of either lncRNA or mediator subunits has been shown to abolish the chromosomal interactions [84].

It has been shown that islet-specific lncRNAs and TFs co-regulate genes associated with enhancer clusters [24, 85]. lncRNAs regulating enhancer cluster-associated genes bound by multiple islet-specific TFs include *HI-LNC12*, *HI-LNC15*, *HI-LNC30*, *HI-LNC78*, *HI-LNC80*, *HI-LNC85*, and *PLUT* (*HI-LNC71)* [24]. However, further studies are warranted that employ chromosomal conformation techniques to identify potential targets of enhancer associated lncRNAs in human islets.

### Future challenges and opportunities

Although lncRNAs have achieved formable recognition as key players in gene regulation and disease, there is still a huge gap in our overall understanding of lncRNA regulatory functions and underlying molecular mechanisms, particularly in context of β cell function and development of T1D. The rise of lncRNAs as key regulators of gene expression during normal development and diseases has positioned these pervasive transcripts in the crosshairs of novel disease-specific biomarker discovery. The dysregulation of lncRNA expression not only represents a newfangled layer of intricacy in the molecular architecture of human malady, but it also unveils the potential to use lncRNAs as disease biomarkers. In contrast to their “cousins” mRNA transcripts, the lncRNAs themselves are functional molecules and their expression levels might serve as a better disease indicator. Moreover, expression of lncRNAs is highly tissue-specific and disease-specific which indicates that lncRNA-based expression signatures could effectively be used to accurately diagnose and classify disease. Although, the field of lncRNA-based diagnostics is still in its infancy, the use of distinct lncRNAs in a clinical diagnostics setting has already taken off. Indeed, lncRNAs have already been suggested as potential biomarkers for a number of diseases, including cancer. For example, *PCA3*, a prostate-specific lncRNA notably overexpressed in prostate cancer, has been developed into diagnostic assay to detect prostate cancer [86]. Nevertheless, there are still many challenges ahead of us that need to be addressed in order to fully appreciate the function of lncRNAs in islet biology and T1D contexts.

Most of the functionally characterized lncRNAs exhibit modular-domain architecture that arises from their well-conserved secondary and tertiary structures and is crucial for their biological functions. This assumes importance as it illustrates the importance of the conservation at secondary and tertiary structure level rather than at primary sequence level [87–89]. Therefore, future studies are needed to identify homologous lncRNAs taking a structure-based evolutionary conservation criterion into consideration rather than relying only on the primary sequence-based conservation in cellular and subclinical models of T1D.

Although, a handful of lncRNAs have been functionally characterized, they have certainly emerged as bona fide players in regulating the gene expression at various levels during all the stages of development and disease. With the advent of next-generation sequencing technologies, thousands of lncRNA genes have already been identified and annotated in human and mouse. According to the recent GENCODE v26 (www.gencodegenes.org), more than 15,000 and 11,000 lncRNA genes have already identified in the human and mouse, respectively. In addition, FANTOM5 cap analysis of gene expression (CAGE) in primary cell types and tissues identified 27,919 human lncRNA genes with accurate 5′ termini [90]. Based on multiple lines of evidence, including genomic, epigenomic features and evolutionary conservation of the lncRNAs, 19,175 were reported as potentially functional in the human genome [90]. Surprisingly, most of the intergenic lncRNAs identified in the FANTOM5 project were transcribed from the enhancers and not from the promoters.

One of the major bottlenecks in studying the lncRNA functions has been their low steady-state levels in the cells [91]. Majority of lncRNAs are expressed at low-levels which makes it more challenging to accurately annotate their gene boundaries. The problem of incomplete annotation of lncRNAs is further compounded by the lack of typical genomic hallmarks of transcription initiation and termination that are often used as primary flag posts for defining the gene boundaries [91]. But, recently many methods have been developed and implemented to improve the annotation of lncRNAs, including RNA Capture Sequencing (CaptureSeq) [92], coupling of rapid amplification of cDNA ends (RACE) technique to long-read sequencing (RACE-Seq) [93], RNA Capture Long Seq (CLS) [94], and genome-scale CRISPR-mediated interference (CRISPRi) [95]. So far, these methods have been used for exploring the transcriptomic structure of lncRNA loci using cell or tissue types that are not relevant to diabetes. Nevertheless, these methods provide an excellent experimental framework for future studies to address the lingering questions regarding the roles and molecular mechanisms of lncRNAs in β cell function and T1D pathogenesis. Therefore, employing methods like CLS, RACE-Seq, CAGE and CRISPRi under the proinflammatory cytokines stimulation and control settings will enable to disentangle the transcriptomic landscape of β cells.

It is known that active β cells exhibit extensive allelic imbalance in gene expression [96]. However, the impact of allelic imbalance on the lncRNA expression has been not studied. A robust reassessment of the regulation of allele-specific gene expression in lncRNA loci overlapping or lying in close proximity of T1D GWAS SNPs in islets and β cells derived from cadaveric pancreas with known genotypes would be highly desirable. This could provide important clues linking disease-associated SNPs with the lncRNA expression. Furthermore, these studies have the potential to unravel the diversity of lncRNA repertoire, including the enhancer RNAs (eRNAs) [97] and novel rare transcripts and many of which might have important functional roles in modulating the β cell function.

## Conclusions

In immune-mediated and inflammatory diseases such as T1D, lncRNA-based transcriptional signatures might open new avenues for lncRNA-based diagnostics, classification or personalized therapeutic regimens in near future. Furthermore, assessment of functional implications of T1D SNPs overlapping lncRNAs and enhancers regions is highly warranted from perspective of β cell function and development of T1D. The precise biochemical characteristics and molecular basis of β- and immune cells expressed lncRNA functions are necessary to elucidate how deregulations of immune cell-specific T1D loci-associated lncRNAs, as well islet-specific lncRNAs, potentially contribute to the development of islet autoimmunity to progression of T1D.

## Abbreviations

eQTL: Expression quantitative trait loci
GWAS: Genome-wide association studies
LD: Linkage disequilibrium
lncRNA: Long non-coding RNA
SNP: Single-nucleotide polymorphism
TF: Transcription factor
TFBS: Transcription factor binding site
T1D: Type 1 diabetes
